# The Risk of Cancer Might be Lower Than We Think. Alternatives to Lifetime Risk Estimates

**DOI:** 10.5041/RMMJ.10321

**Published:** 2018-01-29

**Authors:** Gilat L. Grunau, Shay Gueron, Boris Pornov, Shai Linn

**Affiliations:** 1 Department of Radiology, University of British Columbia, Vancouver, British Columbia, Canada; 2 Department of Mathematics, University of Haifa, Haifa, Israel; 3 Department of Environment Management, University of Haifa, Haifa, Israel; 4 School of Public Health, University of Haifa, Haifa, Israel

**Keywords:** Breast cancer, cancer, epidemiology, lifetime risk, methodology, risk

## Abstract

**Background:**

Estimates of lifetime cancer risk are commonly used in the clinical setting and in health-care evaluations. These measures are based on lifetime cancer risk estimates and may create an unrealistically frightening perception of cancer risk for an individual. We suggest using two new measures of cancer risk to complement the cancer lifetime risk measure, namely estimates of cancer risk from birth to a specific age or from a specific age to life expectancy.

**Methods:**

We calculated risks using incidence density data from the Israel National Cancer Registry of 2013, applying a well-known formula for calculating risk, for a follow-up time. The joint disease-free survival probability is calculated for several age intervals, and hence the risk (i.e. 1–survival) for the intervals.

**Results:**

The risk of cancer to age 80 in Jewish men and women, respectively, ranged from about 0.336 and 0.329 at age 0, to 0.279 and 0.237 at age 60. The risk of cancer from birth up to an age in Jewish men and women, respectively, ranged from 0 and 0 at birth to 0.088 and 0.129 at age 60. The risk of cancer to age 80 in Arab men and women, respectively, ranged from 0.298 and 0.235 at age 0 to 0.249 and 0.161 at age 60. The risk of cancer from birth up to an age in Arab men and women, respectively, ranged from 0 and 0 at age 0 to 0.074 and 0.095 at age 60. In Jewish and Arab women, breast cancer risk to age 80 decreased from about 0.127 in Jewish women at age 40 to 0.079 at age 60 and from 0.080 to 0.043 in Arab women; the risk from birth up to a specific age ranged between 0 and 0.056, and 0 and 0.040, respectively.

**Conclusion:**

The two proposed new estimates convey important additional information to patients and physicians. These estimates are considerably lower than the frequently quoted 33% lifetime cancer risk and are more relevant to patients and physicians. Similarly, breast cancer risk estimates up to or from a specific age differ considerably from the frequently quoted lifetime risk estimates of 1 in 8 women.

## INTRODUCTION

Cancer risk estimates can potentially be easily misunderstood and create an unrealistically frightening perception of risk.^1^ Cancer risk estimates based on the risk in the entire lifespan are commonly quoted in scientific and popular publications. In this paper, we suggest using the following two new measures: (1) An estimate of cancer risk *from a specific age to life expectancy*; and (2) An estimate of cancer risks *from birth up to a specific age*. We use the cancer registry statistics in Israel to calculate these two new measures and compare them with the traditional cancer lifetime risk measure.

### Limitations of the Lifetime Risk Measure

The Surveillance, Epidemiology, and End Results (SEER) Program of the National Cancer Institute (NCI) regularly publishes lifetime risks of being diagnosed with each type of cancer.^2^ According to these estimates, the lifetime risk of all invasive cancers in the USA is 42.05% and 37.58% for men and women, respectively. The lifetime risk of breast cancer in women is 12.32%, i.e. the odds are about 1 in 8.^3^,^4^ The SEER program also estimated the risk of being diagnosed with cancer in 10, 20, and 30 years (from any age). However, SEER^2^ has not offered an alternative to lifetime risk estimate until life expectancy.

Ahmad et al.^5^ estimated that one in two people born after 1960 in the UK will be diagnosed with some form of cancer during their lifetime. They reported that the lifetime risk of cancer in the UK increased from 38.5% for men born in 1930 to 53.5% for men born in 1960, while for women it increased from 36.7 to 47.5%. The publications of the Israel National Cancer Registry (INCR)^6^ also use the “lifetime risk of cancer” as a measure of cancer morbidity.

These publications estimate that 1 in 3 persons in Israel will be diagnosed with cancer in their lifetime. Similar calculations estimated that 1 in 8 women in Israel will be diagnosed with breast cancer in their lifetime. It should be noted that these estimates of lifetime cancer risk indicate only the probability (risk) of being diagnosed with cancer at birth (age 0) through to the end of life, where life expectancy is estimated to be 80 or 90 years, and thus have several limitations:

Specifically, the two main limitations of this measure are as follows.

1. *Lifetime risk does not indicate the future risk of cancer from a specific age to life expectancy.* However, the *future* cancer risk is frequently a focus of interest for individuals: what is the risk that I will have cancer in the future? The answer to this question may change an individual’s personal decisions, as well as financial planning and insurance choices.
2. *Lifetime risk does not indicate the past risk of cancer from birth up to a specific age*, i.e. it does not estimate the risk of being diagnosed with cancer in the past up to any specific age. However, the past cancer risk is frequently a focus of cancer cluster investigations and tort litigations when individuals claim that their cancer rate is higher than expected.^2^

## METHODS

### Measures of Disease Incidence

The cumulative incidence rate (CIR) is a commonly used measure of disease risk, estimated as the proportion (percent) of a population at risk that will develop an outcome in a given period of time.^7^–^15^ It is a measure of risk (i.e. probability) of a disease.

The CIR is most commonly used for estimating risk from the follow-up data of a fixed closed cohort. The numerator for CIR is the number of newly detected cases, and the denominator is the number of disease-free subjects at the beginning of the follow-up. This fraction is a proportion estimation of a probability or risk of disease. Hereafter, we use the term risk (*R*), instead of CIR for simplicity.

Incidence density (ID) has the same numerator as the risk, that is, the number of newly detected cases, but the denominator is the sum of the person-time experience of the at-risk population.^7^–^13^ It is usually calculated in a dynamic open population. Incidence density is an estimate of the instantaneous rate of disease, i.e. the rate at which new cases are occurring at any particular moment. The ID rate is therefore more analogous to the speed of a car, i.e. it measures the rate at which new cases of disease occur per unit of time.

The calculation of the ID assumes that the population is *dynamic and stable* over the follow-up period. Stability implies that the study population’s size and age distribution, and, thus, that of other characteristics, remain constant. Therefore, the computation must generally involve small age categories, because the underlying assumption of the analysis is that the (instantaneous) rate of cancer remains constant over the age/time follow-up period. The INCR uses five-year age categories.

We used ID data to calculate the risks in order to estimate the risk of cancer from birth to a specific age and from a specific age to life expectancy.

### Source of Data

We used the INCR data for 2013,^6^ which are ID data. These data are published for four gender and ethnic categories: men and women, Jews and Arabs. The registry data in a country’s dynamic population comprise ID data for one year of follow-up. The ID data are usually presented for 100,000 person-years, for each five-year age group, assuming that the ID is stable throughout the quinquennial age groups (in a follow-up of one year). This implies that, as a person ages during these five years, the ID remains constant. To demonstrate our methodology, we use the INCR data for 2013 for all cancers and for breast cancer.

### Calculating Risk from ID Data

The risk (*R*) of cancer is frequently calculated from the cancer registry ID rate,^13^ using a well-known formula^10^ for calculating risk, for a follow-up time Δ*t*:

(Eq. 1) $$R_{t_{0},t_{1}}=R_{\Delta t}=1-e^{-ID*\Delta t}$$

When a person is followed up over several j age categories that have a different ID, denoted by *ID_j_*, a summary of the person-time experience is used in the calculations. This constitutes the average risk for a person in that age group. (S represents the sum over the *j* age categories.)

(Eq. 2) $$R_{t_{0},t_{j}}=1-e^{-\sum_{j}ID_{j}*\Delta t}$$

Note that Δ*t* refers to the follow-up time, not to the range of each age stratum. Failure to dissociate these two different periods would yield incorrect risk estimates. For example, the follow-up time of the annual cancer registry reports is one year, while the range of the age categories is five years, e.g. 40–44 years of age.

The use of these equations involves known limitations and assumptions.^10^,^13^ The equation assumes a constant ID for the follow-up period, which is a logical assumption for one year of follow-up in a narrow range of age stratum, and also assumes no competing risks.

### Using Age-specific ID for Calculating Risk for a Given Follow-up Period

Using the notations of Rothman et al.,^13^ we can write that the *disease-free survival* (no disease occurrence) probability *S* after the follow-up *k* year, for the *j^th^* age category, is 1–risk in that year. Thus, for the first age category we can use

(Eq. 3) $$\begin{array}{c} S_{1}=1-R_{1}=1-1+e^{-ID_{j}*\Delta t_{k}}\\ =e^{-ID_{1}*\Delta t_{k}} \end{array}$$

Therefore, for a person in each age in the first age category the survival is

(Eq. 3.1) $$\begin{array}{c} S_{1}=1-R_{1}=1-1+e^{-ID_{j}*\Delta t_{k}}\\ =e^{-ID_{1}*\Delta t_{k}} \end{array}$$

Disease-free survival in the second age interval is conditioned on the survival in the first age interval. The joint survival probability in the first age interval and the second age interval is

(Eq. 4) $$S_{1,2}=S_{1}S_{2}$$

so that

(Eq. 4.1) $$S_{1,2}=S_{1}S_{2}=(1-R_{1})(1-R_{2})$$

Assuming similar follow-up time D*t_k_*, we obtain

(Eq. 5) $$\begin{array}{c} R_{1,2}=1-S_{1}S_{2}\\ =1-(1-R_{1})(1-R_{2})\\ =1-e^{-ID_{1}*\Delta t_{k}}e^{-ID_{2}*\Delta t_{k}}\\ =1-e^{-\Sigma_{1}^{2}-ID_{j}*\Delta t_{k}} \end{array}$$

In general, the risk for the 1 … *j^th^* age category (using P for multiplication) is

(Eq. 6) $$\begin{array}{c} S_{1..\text{j}}=\Pi (1-R_{1})\ldots (1-R_{j})\\ =e^{-\Sigma_{1}^{j}-ID_{j}*\Delta t_{k}} \end{array}$$

And thus,

(Eq. 7) $$\begin{array}{c} R_{1..\text{j}}=1-S_{1..\text{j}}\\ =1-\Pi (1-R_{1})\ldots (1-R_{j})\\ =1-e^{-\Sigma_{1}^{j}-ID_{j}*\Delta t_{k}} \end{array}$$

Note that follow-up times may not be uniform and may also be different for each age category. For simplicity, we assume equal follow-up times, i.e. D*t_k_* is constant. The INCR data are always for one year of follow-up, per 100,000 persons, for each age category, i.e. ID data per 100,000 person-years. If the follow-up time is D*t_k_*=1 year (e.g. as in the yearly report of a cancer registry), the equation is

(Eq. 8) $$\begin{array}{c} R_{1..\text{j}}=1-\Pi (1-R_{1})\ldots (1-R_{j})\\ =1-e^{-\Sigma_{1}^{j}-ID_{j}*1}=1-e^{-\Sigma_{1}^{j}-ID_{j}} \end{array}$$

### Age-specific Risk Calculations

Using the above equations, we calculated the risk for any follow-up period from the age-group-specific risks using ID data. Then, we calculated the survival rates (i.e. 1−*R*) for specific follow-up times. Next, we calculated the age-specific risk of cancer, in the following steps. The relevant age group’s ID was used as the age-specific ID. Thus, for example, the 20–24 age-group-specific ID was used as the age-specific ID for the ages 20, 21, 22, 23, and 24 years. In this sense, we followed the INCR practice of assuming that the ID was stable within each age group. Each ID (rate of cancer per 100,000 person-years in 2013) represents a follow-up time of one year for each age within the age group range.

Age-specific risks were calculated for each age in an age group interval (i.e. for each age 20, 21, 22, 23, and 24 years for the 20–24 year age group interval) using Equation 2. Age-specific cancer-free survival probabilities were calculated for each age using Equation 3; i.e. for each of the five years of the follow-up we used the relevant age-group-specific ID to calculate the age-specific survival probabilities for each age.

Cumulative cancer-free survival probabilities for a range of ages were calculated by multiplications of the age-specific survival probabilities in the range using Equation 6.

The risks for a range of ages were calculated using Equation 8.

### Calculation of the Two New Measures for All Cancer Types

We calculated two risk estimates that address the two issues stated in the introduction section as follows:

1. *The future risk from a specific age up to life expectancy* was calculated for a person from a specific age up to a life expectancy of 80 years, using ID data after the specific age up to age 80. We assumed a follow-up time of one year at each age, throughout the remaining years up to life expectancy as a person ages and becomes a member of other age categories. We also assumed that the 2013 ID data for each age category are stable estimates for future years, as a person ages. For example, for a person aged 20, we multiplied the calculated risk for each age from age 20 until age 80, based on the age-group-specific ID (ID was assumed stable for each age category). The cancer-free survival probability is calculated using the above Equations 3–6. The risk of a person aged 20 years can be calculated for 60 years until life expectancy (80 years) using the above Equations 7 and 8. As the 20-year-old person is aging and becoming a member of all age categories until age 80, the cumulative risk is calculated by multiplying the age-specific cancer-free survival probabilities, and the total risk would be 1–cumulative survival, as explained above (Eq. 8).
  These calculations provide an estimation of the risk of being diagnosed with cancer from a specific age (e.g. 20 years in the example) at any time in the future after 20 years of age until life expectancy.
2. *The past risk from birth (age 0) to a specific age* was calculated for a person from birth until a specific age. We used ID data from age 0 until that age. For example, for a person aged 20 years cumulative cancer-free survival probabilities were calculated by multiplying the cancer-free age-specific probabilities from birth until age 20. The risk for ages 0 to 20 (i.e. 1–cumulative survival probabilities) was then calculated. In other words, as this person ages from age 0 to age 20, the total risk would be the 1–multiplication of the specific age survival probabilities (Equation 8).

### Calculation of the Two New Measures for Breast Cancer

Similarly, we calculated the three new estimates of breast cancer risk using the INCR data for 2013.

## RESULTS

**Figure 1:**
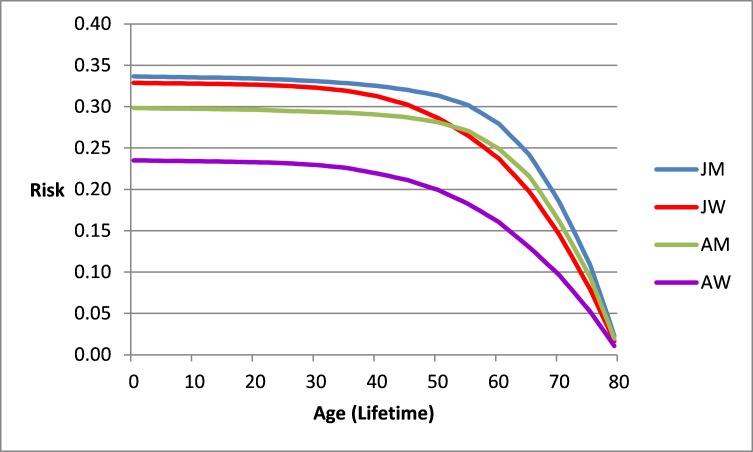
Risk of Any Cancer from an Age up to Age 80 in Jewish Men (JM), Jewish Women (JW), Arab Men (AM), and Arab Women (AW) in Israel. Israel National Cancer Registry data 2013.

**Figure 2:**
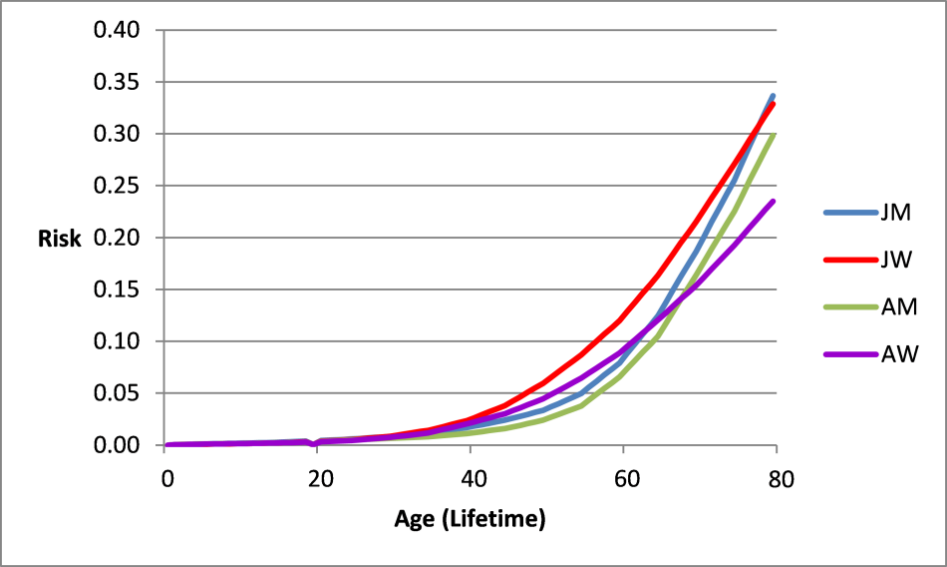
Risk of Any Cancer from Birth to an Age in Jewish Men (JM), Jewish Women (JW), Arab Men (AM), and Arab Women (AW) in Israel. Israel National Cancer Registry data 2013.

**Figure 3:**
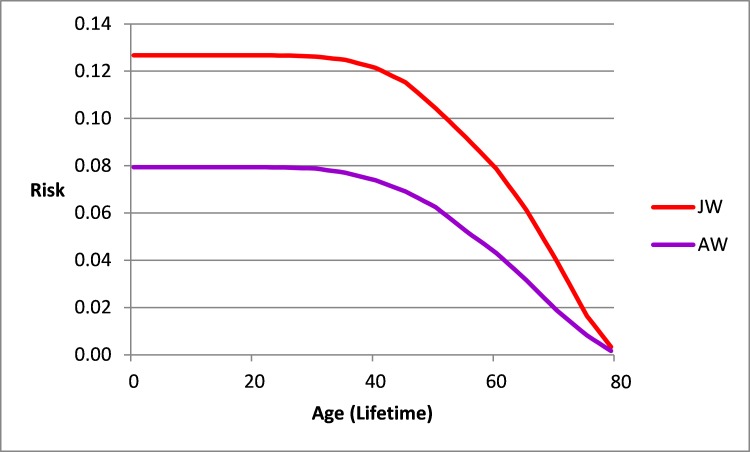
Risk of Breast Cancer from a Specific Age up to Age 80 in Jewish Women (JW) and Arab Women (AW) in Israel. Israel National Cancer Registry data 2013.

**Figure 4:**
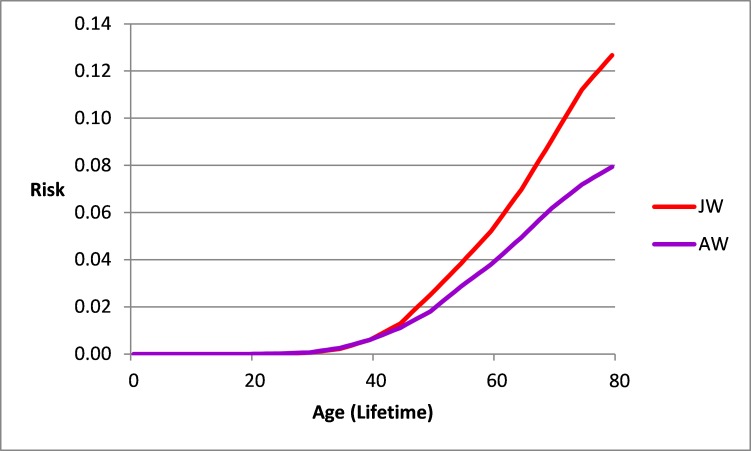
Risk of Breast Cancer from Birth up to an Age in Jewish Women (JW) and Arab Women (AW) in Israel. Israel National Cancer Registry data 2013.

For simplicity and readability, the results (Tables 1–3, Figures 1–4) are presented for selected ages: 0, 20, 40, and 60 years for all cancers, and 0, 20, 40, 50, and 60 years for breast cancer. For breast cancer, we chose to include the calculated risk measures for age 50 because of the common practice of offering mammography to women at age 50 and over.

**Table 1:** All Cancer Rates in 2013 for Jewish Men and Women; Risk for Follow-up from a Specific Age Up to Age 80; and Risk from Birth Up to a Specific Age.

Age	Jewish Men			Jewish Women		
	Rate per 100,000 Person-years	Risk from an Age to Age 80	Risk up to an Age	Rate per 100,000 Person-years	Risk from an Age to Age 80	Risk up to an age
0	18.49	0.336	0.000	14.74	0.329	0.000
20	37.37	0.334	0.004	40.96	0.327	0.004
40	147.73	0.325	0.018	299.61	0.313	0.026
60	1006.20	0.279	0.088	1014.50	0.237	0.129

**Table 2:** All Cancer Rates in 2013 for Arab Men and Women; Risk for Follow-up from a Specific Age Up to Age 80; and Risk from Birth Up to a Specific Age.

Age	Arab Men			Arab Women		
	Rate per 100,000 Person-years	Risk from an Age to Age 80	Risk up to an Age	Rate per 100,000 Person-years	Risk from an Age to Age 80	Risk up to an Age
0	20.65	0.298	0.000	16.55	0.235	0.000
20	38.81	0.296	0.003	29.65	0.233	0.002
40	94.26	0.290	0.012	202.48	0.219	0.022
60	854.55	0.249	0.074	709.50	0.161	0.095

**Table 3:** Breast Cancer Rates in 2013 for Jewish and Arab Women; Risk for Follow-up from a Specific Age Up to Age 80; and Risk from Birth Up to a Specific Age.

Age	Jewish Women			Arab Women		
	Rate per 100,000 Person-years	Risk from an Age to Age 80	Risk up to an Age	Rate per 100,000 Person-years	Risk from an Age to Age 80	Risk up to an Age
0	0	0.127	0	0	0.080	0
20	1.45	0.127	0	44.04	0.079	0
40	139.30	0.121	0.007	103.31	0.073	0.007
50	271.15	0.104	0.028	214.29	0.065	0.020
60	377.68	0.079	0.056	240.22	0.043	0.040

### Risk of All Cancers

Table 1 describes the 2013 INCR data for Jewish men and women for ages 0, 20, 40, and 60 years.

For Jewish men, the risk of cancer from these ages to age 80 ranged from 0.336 at age 0, and decreased to 0.279 for age 60. The risk of cancer from birth up to an age ranged from 0 at birth (as expected) to 0.088 at age 60. Similar but slightly higher risks from an age to age 80 were found for Jewish women. However, the risks up to an age for Jewish women were higher after age 40.

Table 2 describes the 2013 INCR data for Arab men and women for ages 0, 20, 40, and 60 years with similar findings. However, the risks of Arab women in all ages were lower than the risks for Arab men.

Figure 1 summarizes the cumulative risk of any type of cancer *in the future* from a specific age to age 80 in Jewish and Arab man and women in Israel as compared to the lifetime risk of 0.33. The risk from a specific age to age 80 is above 33% until age 31 years for Jewish men and declines thereafter. It is always below 33% for Jewish women, and for Arab men and women.

Figure 2 summarizes the risk of any type of cancer *in the past* from birth up to a specific age in the four ethnic and gender strata, as compared to the lifetime risk of 0.33. The risk up to an age is above 33% only at age 79 for Jewish men and women. The highest risk for Arab men and women is lower and reaches 0.30 and 0.24 for Arab men and women at age 79, respectively.

### Risk of Breast Cancer

Table 3 describes the 2013 INCR breast cancer data for Jewish and Arab women for the ages 0, 20, 40, 50, and 60 years. The breast cancer risks of Arab women are in general lower than those of Jewish women.

The risk in the future up to age 80 decreased from 0.127 in Jewish women at age 40 and younger (similar to the commonly published “1 in 9” estimated odds) and decreased thereafter to 0.079 at age 60. Lower rates were found for Arab women, with a range between 0.080 and 0.043. The past risk from birth up to a specific age ranged between 0 and 0.056 for age 60 in Jewish women and from 0 to 0.040 in Arab women.

Figure 3 describes the risk of breast cancer in the future from a specific age to age 80, as compared to the frequently quoted breast cancer lifetime risk of 0.125. Until age 45, the risk is above 0.120 and 0.080 among Jewish and Arab women, respectively, and thereafter decreases.

Figure 4 describes the risk of breast cancer in the past from birth up to a specific age in Jewish women and Arab women in Israel, as compared to the frequently quoted breast cancer lifetime risk of 0.125. The risk is below 0.02 among both Jewish and Arab women up to age 45 and 49, respectively. Thereafter, the risk increases more quickly among Jewish women, and reaches approximately 0.120 only after age 75. The highest risk among Arab women is 0.080 at age 80.

## DISCUSSION

The frequently reported “lifetime risk” of cancer statistics has two main limitations: it may not indicate the important statistics that may be the main concern of patients and physicians, i.e. the cancer risk *in the past* up to a certain age, and *in the future* from a specific age to life expectancy. We can suggest “cancer risk in the past risk (CRIP)” and “cancer risk in the future (CRIF)” as names for the suggested new measures.

In this paper, we propose two new measures that can be used to address these issues of interest. These measures can easily be calculated from the published statistics of national registries and are applicable for individuals at any age. The proposed new measures are clinically oriented. They provide information that may be useful to patients and physicians, and can be interpreted and used straightforwardly.

Our analyses indicate that relevant cancer risk estimates from birth to a specific age, or from a specific age to life expectancy, or the average risk in a specific age group, are all considerably lower than the frequently quoted 33% lifetime cancer risk. Similarly, breast cancer risk estimates at specific ages are different from the frequently quoted estimated odds of 1 in 8 or 1 in 9 women. These lower cancer probabilities have important implications for individuals, and also for the public health system in terms of predicting the health resources required for cancer patients.^2^,^7^,^12^ These two estimates are also more informative than the lifetime risk estimates and more relevant to individuals at any specific age.

The *future* cancer probability *from* a specific age could be used to assess an individual’s probability of being diagnosed with cancer in the future, and the probability can be modified by considering risk factors such as smoking, family history, etc. This statistic may have implications on a person’s future productivity, potential insurance calculations, and the public health system’s estimates of medical services allocation and planning, per each age.

The *past* cancer probability *up to* a specific age could be used to assess the excess of cancer occurrence in populations and individuals, in litigations, occupational settings, and toxic tort litigations.^16^ For example, the present cancer probability of a person who has been diagnosed with cancer could be compared with that of having cancer in the past up to his/her present age. In toxic tort litigations, for a group of persons who have been diagnosed with cancer, the median age of diagnosis could be compared to the probability of cancer until that age in the general population, based on the cancer registry.

Bender et al.^17^ noted the limitations of the lifetime risk measure and suggested new population measures, that is, the cumulative risk for an entire cohort, using lifetable methodology, to estimate a quantitative index of the cancer burden in the community (e.g. a state). They indicated that their new measure estimates the population cancer risk (PCR) and expresses it as the number of expected cancers in the lifetimes of 1,000 people. This index has applications in program planning, communicating the risk of cancers, and descriptive epidemiology. Our measures are applicable for individuals, rather than populations, as suggested by Bender et al., and may be more informative in clinical settings and thus complement the methodologies mentioned above.

Our methodology is based on published registry data and thus is available to any person or health professional who can access published registry data. Because it uses registry data, it has the same limitations as all registry data, including incomplete ascertainment, possible selection and information bias, or bias due to multiple malignancies in the same person.^18^

In addition, our calculations in Equations 1–8 are based on specific assumptions, as mentioned, i.e. stable ID in the relevant period and an absence of competing risks. In the calculations, we assumed that the ID for each age group in the past or the future is identical to that in 2013. Future research could attempt to adjust for the rate of change in cancer rates throughout the relevant year periods. However, this limitation in our methodology is similar to any lifetime cancer risk calculations that are currently used. Similar to the commonly used “lifetime risk” measure from birth to life expectancy, our new measures give identical weight to all ages and all age-specific cancer rates. Future research could attempt to standardize these measures, or develop further measures of years of potential life lost based on our suggested measures, as has been suggested by Sasieni and Adams.^19^

Following SEER,^2^ we suggest that future research could use our new measures to calculate the risk from any age 10, 20, 30, 40, 50, etc. years ahead, similar to our calculations to age 80 (life expectancy).

Further research could explore the use of our suggested measures: clinicians in one clinic could use these new estimates, and clinicians in a comparable clinic could use the old estimates when talking to their patients, and the pre–post attitudes and behaviors of patients belonging to the two clinics could then be compared. The results could serve as a measure of the feasibility, efficacy, and effectiveness of our suggested new measures, as in other research on other health measures.

We used 80 as an estimate of life expectancy; however, our calculations should be modified for countries or populations with a different life expectancy.^2^

A limitation of our study is that data for only one year (2013) were used. We used these data as these were the most recent available data of the INCR, and for demonstrating the use of our suggested new measures. Thus, our estimates of risks are limited to this year only. Future research could compare these estimates with those for other years.

Our analyses indicate that relevant cancer risk estimates from birth to a specific age, or from a specific age to life expectancy, are all lower than the frequently quoted lifetime cancer risks. Our estimates may be more informative than the currently used lifetime risk measure, which may overly alarm individuals or decision-makers.^1^ For example, Hopwood^20^ indicated that women’s perceptions of breast cancer risk are largely inaccurate and are frequently associated with high levels of anxiety about cancer. Further research could explore the use of our measure in communicating cancer risk. Our age-specific estimates of cancer risks may have important implications in public health settings and in predicting the necessary health resources for cancer patients.

In summary, we suggest that the new measures could complement the published registry “lifetime risk” statistics and potentially provide more relevant information to patients and physicians.

## Abbreviations

CIR: cumulative incidence rate
ID: incidence density
INCR: Israel National Cancer Registry
NCI: National Cancer Institute
R: risk
SEER: Surveillance, Epidemiology, and End Results
